# Biological and Mechanical Factors and Epigenetic Regulation Involved in Tendon Healing

**DOI:** 10.1155/2023/4387630

**Published:** 2023-01-09

**Authors:** Zhi Jie Li, Qian Qian Yang, You Lang Zhou

**Affiliations:** Hand Surgery Research Center, Research Central of Clinical Medicine, Affiliated Hospital of Nantong University, Medical School of Nantong University, Nantong 226001, China

## Abstract

Tendons are an important part of the musculoskeletal system. Connecting muscles to bones, tendons convert force into movement. Tendon injury can be acute or chronic. Noticeably, tendon healing requires a long time span and includes inflammation, proliferation, and remodeling processes. The mismatch between endogenous and exogenous healing may lead to adhesion causing further negative effects. Management of tendon injuries and complications such as subsequent adhesion formation are still challenges for clinicians. Due to numerous factors, tendon healing is a complex process. This review introduces the role of various biological and mechanical factors and epigenetic regulation processes involved in tendon healing.

## 1. Introduction

Tendons are the connective tissue that links muscles to bones. They are highly resistant to mechanical loads. Tendons transmit, distribute, and regulate the force applied by muscles to connective structure and anchor muscles [1, 2].

Mature tendons are comprised of tightly packed collagen fibers of different sizes. Tendon cells are distributed between fibers and synthesize a lot of extracellular matrices (ECMs), composed mostly of collagen and proteoglycan, which lubricates and assembles collagen fibers [3]. Despite having a high tensile strength and the ability to endure the powerful forces, produced by skeletal muscle contractions, tendons are susceptible to acute or chronic injury [4]. There are several biological causes of tendon injury including trauma, chronic overuse, aging, inflammation, and genetic factors [5–8]. Physical factors such as acute mechanical loads often lead to tendon tears. Clinical symptoms of chronic tendon injury or tendinopathy include pain, decreased muscle strength, and decreased physical activity [9].

Tendon injuries are common causes for seeking medical attention and account for 30% of all musculoskeletal symptoms [10]. Since connective tissue is different from other highly vascularized tissues, its sparse vascular network and low cellular metabolic rate lead to poor intrinsic healing of tendon resulting in restricted tendon regeneration potential. The healing of injured tendons needs a long time [11–13]. To make matters worse, complications often linger on even after a long recovery period. Noticeably, the formation of tendon adhesions remains the leading cause of disability in all instances of complications. When tendons are injured, they often form disordered scar tissue that does not heal well. Such restrictive adhesions within the synovial sheath of the tendon can impair function, increase the rerupture risk, and severely hinder tendon healing [14–16].

Inflammation, proliferation, and remodeling are three overlapping stages of tendon healing. The intrinsic tendon cells from inside the tendon along with the external and peripheral fibroblasts cooperate during the healing process [17]. Inflammation is the first stage of tendon healing and it involves inflammatory cells and the invasion of exogenous fibroblasts to the injury site. During the inflammation stage, a large number of cytokines are secreted by inflammatory cells. These cytokines stimulate cell migration and neovascularization. The second stage is the repair process during which numerous regenerative activities are carried out under the guidance of tenocytes and macrophages. These activities include fibroblast proliferation, excessive ECM synthesis, tendon stem/progenitor cell (TSPC) activation, and tendon differentiation. Finally, new collagens reassemble to form healing tissue, with type I collagen playing a leading role [18].

Stem cells possess the ability to expand and differentiate into various cell lines. Stem cells also participate in tendon regeneration by regulating inflammation and promoting scar tissue regeneration. Tendon stem/progenitor cells (TSPCs) are an ideal cell type as the largest number of tendon-related markers is noticed during TSPC expression. Noticeably, elderly people possess fewer TSPCs than those in younger adults, which may explain the high incidence of tendinopathy in the elderly population. Importantly, both in vitro as well as animal studies have reported that TSPCs do differentiate into tendon cells [9].

Recent studies have reported that tendon adhesion is caused by an imbalance between endogenous healing pathways led by tenocytes and fibroblast-led exogenous healing pathways [19, 20]. The proliferation and differentiation of tendon cells primarily support endogenous healing by preventing tendon adhesion. However, exogenous healing involves the proliferation of fibroblasts around the tendon leading to growth at the broken end of the tendon which eventually leads to scar tissue formation. Often, fibroblast activity causes aggregation of collagen and fibrin around the site of injury causing adhesions, which in turn, reduce the affected joint's range of motion. Therefore, tendon adhesion is inevitable in cases where exogenous healing is more prominent than endogenous healing. Other causes for the formation of tendon adhesion include the destruction of the blood supply, loss of integrity of the tendon, and the inflammatory reaction of the tissue around the tendon. Moreover, fibrin leakage exacerbated by inflammatory exudates further worsens adhesion [16, 18].

Currently, conservative and surgical treatments are the two primary methods for treating tendon and ligament injuries. While the conservative treatment involves daily rest, drug administration, or ice administration, the surgical treatment has a restorative effect [21, 22]. Despite significant advances in surgical and rehabilitation methods, functional recovery remains limited when dealing with cases involving gap formation, adhesion, and rupture. Consequently, the regenerative capacity of tendon is insufficient to fully return to the preinjury condition [23]. Thus, researchers have been trying to improve tendon repair methods which have led to the development of advanced tendon healing methods such as gene therapy, stem cell therapy, platelet-rich plasma (PRP) therapy, growth factors, drugs, and tissue engineering [9].

**Figure 1:**
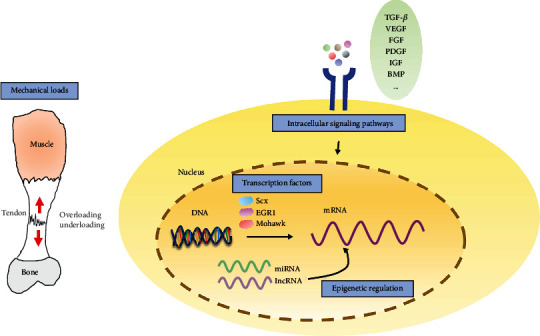
Biological and mechanical factors and epigenetic regulation involved in tendon healing. EGR1: early growth response 1; Scx: scleraxis; TGF-*β*: transformational growth factor-beta; VEGF: vascular endothelial growth factor; FGF: fibroblast growth factor; PDGF: platelet-derived growth factor; IGF: insulin-like growth factor; miRNA: microRNA; lncRNA: long noncoding RNA.

In this review, the effects of biological and mechanical factors and epigenetic regulation on tendon healing are discussed. Further, we examine the relationships between biological, mechanical, and epigenetic factors to provide theoretical support to more effectively address the tendon-healing process (Figure 1 and Table 1).

## 2. Biological Mediation of Tendon Healing

Tendon injury can lead to a series of biological changes in the body, and these complex changes are related to the signaling pathways involved in each process.

Oxygen and lactic acid levels strictly regulate the regenerative activity of the healing tendon. Macrophages indirectly promote collagen formation by releasing large amounts of lactate [24]. Additionally, hypoxic conditions and elevated lactate concentrations stimulate angiogenesis [25]. These physiological changes may be related to the effect of oxygen on some biological factors, such as transformational growth factor-beta (TGF-*β*) [24].

Matrix metalloproteinases (MMPs) are enzymes that depend on zinc to function and participate in tendon healing [26, 27]. While MMP-9 and MMP-13 contribute to the degradation of the ECM several days after tendon injury, MMP-3, MMP-4, and MMP-14 are involved in matrix degradation and matrix remodeling throughout the healing process [28, 29].

Growth and transcription factors as well as some proteins have an important impact on tendon healing, and researchers have investigated these factors with some good results over the last 15 years [9]. According to recent advances, tissue engineering and gene therapy in tendon therapy often involve these factors. For example, 3D printing technology was used to implant tendons loaded with TGF-*β*1 gene silencing plasmids having sustained release and good biocompatibility with chicken tendon injury models [30].

### 2.1. Growth Factors

#### 2.1.1. Transforming Growth Factor-Beta (TGF-*β*) Signaling

There are currently three main subtypes of TGF-*β* that participate in many cellular pathways [31]. TGF-*β*1 is considered to take part in initial inflammatory responses, angiogenesis, collagen synthesis, and fibrosis or excessive scarring during tendon healing [32–34]. Meanwhile, TGF-*β*2 and TGF-*β*3 are considered to be indispensable factors for tendon development and can induce the differentiation of tendon stem cells [35, 36]. These TGF-*β* ligands mainly mediate biological responses through the intracellular Smad2/3 pathway [33, 37–39]. Additionally, in terms of gene expression levels, the application of TGF-*β*3 to tendon cells can increase the level of Smad7 and reduce the level of Smad3, thereby reducing exogenous scarring and tendon adhesion, thereby promoting tendon repair [40]. Similarly, another study demonstrated that suppression of Smad3 may play a role in rotator cuff repair [41].

However, the role of TGF-*β* is also controversial. It can promote the differentiation of fibroblasts, but at high concentrations, TGF-*β* cannot play this role and may even induce apoptosis [42]. Exogenous transmission of TGF-*β* has long been a therapeutic approach studied in vitro and in vivo. TGF-*β*1 is thought to cause excessive scarring. TGF-*β*1 upregulation has been linked to the rising deposition of fibronectin and type I and III collagen, confirming that tendon repair through scar tissue is the basis of tendon adhesion formation [43–45]. In vitro, TGF-*β*1 treatment of tenocytes enhanced ECM and decreased matrix remodeling MMPs, which suggests that it may stimulate the development of adhesions [46]. Bone marrow mesenchymal stem cells transfected with TGF-*β*1 cDNA significantly improved the biomechanical properties of rabbit Achilles tendon injury [47]. Studies have shown that TGF-*β*1 inhibition can decrease the degree of scar formation, but the biomechanical power of tendon repaired sites is reduced. Unlike TGF-*β*1, ectopic delivery of TGF-*β*3 has shown excellent promise. TGF-*β*3 promoted tendinous differentiation of stem cells in coculture [48]. Extrinsic delivery of TGF-*β*3 improved the tendon structure and mechanical properties after Achilles tendon injury in rats [49].

Due to these conflicting roles that TGF-*β* signaling plays in tendon scar formation, the direct effect of TGF-*β* signaling in tendon regeneration is unclear. There are many studies that try to address this ambiguity. For example, Kaji et al. [50] compared tendon healing with and without TGF-*β* and found that scleraxis- (Scx-) tenocyte proliferation and subsequent tenoblast recruitment and functional recovery depended on a TGF-*β* signal whereas Scx+tenocyte early proliferation did not [50].

#### 2.1.2. Vascular Endothelial Growth Factor (VEGF) Signaling

Angiogenesis is an early event in tendon healing that causes many cells, such as fibroblasts, to converge on the area of injury to assist in tendon healing. The newly formed blood vessels mainly remove waste, deliver oxygen and nutrients, and transport biological factors. This is also the role of neovascularization in tendon repair [51–54]. Additionally, dysplasia is understood to inhibit normal healing of tendons after injury and further impair tendon quality [55]. The proliferation of vascular endothelial cells and perivascular cells is conducive to neovascularization, and VEGF has also been shown to stimulate the self-renewal ability of these cells, assist in angiogenesis, and improve the permeability of the neovascularization wall [56–59]. Additionally, VEGF can stimulate fibroblast multiplication and promote leukocyte chemotaxis [60, 61].

Proteins in the VEGF family include VEGFA, VEGFB, VEGFC, VEGFD, placental growth factor (PLGF), VEGFE encoded by a virus, and VEGFF derived from snake venom. Binding to VEGF receptors (VEGFRs), VEGFA is a typical member of the family and the most effective stimulator of angiogenesis [56, 62].

The expression of VEGF changes following acute and chronic tendon injuries. Oxygenation, inflammation, mechanical stress, and nerve signaling can affect VEGF expression levels [63–66]. First, tissue hypoxia induced by tendon injury promotes hypoxia-inducible factor-1 (HIF-1) expression and in turn stimulates VEGF gene expression. Second, the synthesis of VEGF is also induced by the liberation of inflammatory cytokines, such as interleukin-1*β* (IL-1*β*), interleukin-6 (IL-6), and interleukin-8 (IL-8). In addition, due to the upregulation of nerve growth factor (NGF), nerve fibers in damaged tendons tended to grow endogenously. Finally, the use of tendons under long-term overstrength mechanical load can also lead to VEGF upregulation in tendon injury [67]. After acute injury, due to an almost complete cessation of blood supply to the tendon, the metabolic rate during healing is elevated, leading to tendon hypoxia [68]. The presence of inflammatory factors after acute tendon injury and inflammation in the damaged area can also promote the upregulation of VEGF [65, 69, 70].

The increase in VEGF can promote the generation of new blood vessels in the early phase of the repair process. Nutrients, surrounding cells, and growth factors are transported to damaged areas in the process of angiogenesis mediated by VEGF [58]. Further, neovascularization involves more pericytes, which surround tendon vasculature and have the ability to self-renew and regenerate and can transform into tendon stem cells, thus mediating tendon injury healing [16, 69–71]. Then, as the blood vessel formation and repair processes are gradually completed, oxygen supply to the damaged area may be improved, and inflammation and VEGF expression may decrease [72, 73]. The periodic changes in VEGF and neovascularization were regarded as indicators of the severity and recovery status of the injury [68, 72].

However, there is some controversy over VEGF's role in promoting tendon angiogenesis. In chronic tendon diseases, VEGF was elevated in the degenerate tendons compared with natural tendons [66, 68, 74], while tendons induce the growth of new blood vessels by secreting angiogenic growth factors; unfortunately, these new blood vessels are useless and do not provide the oxygen and nutrients needed to reverse hypoxia. Since tissue regeneration requires an adequate supply of oxygen and nutrients, the presence of neovascularization in tendinopathy can be considered a sign of persistent hypoxia and a failed attempt at tissue repair. Since hypoxia-induced new blood vessels are hyperpermeable, these new blood vessels do not provide the oxygen and nutrients needed for tissue maintenance and regeneration. Moreover, the high permeability also explains an apparent paradox as to why there is persistent hypoxia in areas of neovascularization [75]. Additionally, in chronic tendinopathy, the microrupture of tendon microvessels caused by chronic tendon load initiates a vascular remodeling cascade mediated by VEGF, leading to the activation of new vessels. Hence, there is evidence that VEGF-induced angiogenesis cannot play a positive role in tendon healing in degenerative tendon diseases and may also have a negative impact on mechanical capability [64, 68, 76]. This negative effect may result from the potential of VEGF to stimulate the expression of MMPs and inhibit the expression of tissue inhibitors of matrix metalloproteinases (TIMPs) in endothelial cells and fibroblasts [64, 66]. This in turn leads to the destruction of type I collagen, a crucial part of the ECM playing an important role in mechanical loading [76].

#### 2.1.3. Fibroblast Growth Factor (FGF) Signaling

FGFs are polypeptide growth factors widely expressed in developing and adult tissues. There are seven members of the FGF gene family in humans; among which, FGF2 was one of the earliest members discovered [77]. As a single-chain peptide that binds to heparin, FGF2 stimulates mitosis and neovascularization [58, 78]. The expression level of FGF2 was greatly increased after tendon injury, and thus, it could be an important aid in tendon repair. FGF2 has been found to play a role in inflammation, angiogenesis, cell proliferation, and collagen synthesis during tendon healing [79–81].

In a rabbit model of acute rotator cuff tear, endogenous FGF2 is secreted by tendon sheath and ligament cells [82]. Further, elevated FGF2 levels occur at an early stage of tissue healing, which leads to many positive effects, including cell proliferation and migration, neovascularization, and collagen synthesis. Consequently, from ligament therapy, exogenous FGF2 is speculated to cooperate with endogenous FGF2 at an early stage of tendon healing. This cooperative effect can facilitate cell multiplication, ECM formation, and ECM remodeling; thus, significantly shortening tendon healing time [83].

FGF2 has been shown to induce upregulation of type I or III collagen mRNA, which is critical in maintaining the mechanical properties of ligaments and tendons. Therefore, possibly, the ultimate improvement in function is associated with the mRNA level upregulation of collagen that is intrinsic to the process [79, 84].

#### 2.1.4. Platelet-Derived Growth Factor (PDGF) Signaling

The PDGF family has four subtypes, including PDGF-BB [85]. The impassability of the PDGF signaling pathway limits the effect of mechanical stimulation on tendon tissue growth in adult mice, which may be why PDGF signaling is critical to tendon homeostasis [86]. In many animal models, exogenous application of PDGF is beneficial for the morphology and mechanics of tendon healing, demonstrating that PDGF can aid tendon regeneration [87–91].

Importantly, the PDGF-BB dimer can bind to a wide range of surface binding proteins. Therefore, PDGF-BB is the universal isomer of PDGF and has been widely studied [13]. The role of PDGF in tendon healing is multifaceted. Following thrombosis, platelets release a variety of growth factors, which interact with one another. Moreover, tendon cells and fibroblasts are drawn to damage sites by PDGF, and there, they start the production of ECM components [92]. PDGF also promotes tendon healing by promoting neovascularization, stimulating tendon cell migration to the wound area, and increasing tendon cell differentiation [13]. Additionally, PDGF contributes to the chemotaxis and proliferation of white blood cells that are capable of the decomposition and clearance of tissue fragments. PDGF promotes tendon cell proliferation, collagen synthesis, and neovascularization, contributing to early tendon repair, which is beneficial to tendon morphology and function in the regeneration process [13].

#### 2.1.5. Bone Morphogenetic Protein (BMP) Signaling

BMPs are multifunctional growth factors belonging to the TGF-*β* superfamily and were originally found to be inducers of bone and chondrogenesis [93]. Further, many members of this large population are known to be important in the formation of organs and tissues before birth [94, 95].

Several laboratories have demonstrated that three types of BMP (BMPs 12-14), named after growth and differentiation factor (GDF) 5-7 in mice and rats, can be inducers of tendon tissue in vivo [96–98]. Specifically, BMP-14 improved tendon healing in mice [99–101] and induced bone marrow mesenchymal stem cells to differentiate into tendons based on increased genes that encode tendon markers such as Scx, tenascin, and type I collagen [102]. BMP-13 may be an effective stimulus for ectopic tendon formation [103]. BMP-12 is also a cell differentiation agent for tendon healing and development in vivo and in vitro through gene transfer or following in vitro exposure [104–106].

Further, BMP-2, BMP-4, BMP-6, BMP-7, and BMP-9 are of great importance in cartilage osteogenesis and are collectively referred to as chondrogenic BMP [107]. Not only does BMP contribute to the creation and development of cartilage but also of tendons. Recent studies suggest that BMP-2 facilitates the differentiation of bone, fat, and cartilage tissues but downregulates the expression of tenogenic markers in tendon-derived stem cells (TDSCs). This process may be related to the chondrogenesis, fat infiltration, and ossification present in tendinopathy [108, 109]. Specifically, due to a reduced supply of TDSCs for tendon development, BMP-2-induced differentiation of TDSCs into nontenocytes may prevent tendon repair. Further, some inhibitors, such as BMP receptor blockers, have the power to stop the chondrogenic pathway and guarantee the differentiation of tenocytes. The effectiveness, dose, and adverse effects of these inhibitors in the management of tenosynovial illness, however, require additional research [9].

#### 2.1.6. Insulin-Like Growth Factor (IGF) Signaling

The IGF family can be divided into two members: IGF-I and IGF-II, which are closely linked with insulin processes [110]. IGF-1 promotes collagen production in fibroblasts, which is associated with the mechanical load of the tendon. However, the relevant signaling pathways remain unclear [111–113]. Evidence also suggests that IGF-I indirectly promotes collagen synthesis via TGF-*β*1 [114, 115].

The liver secretes IGF influenced by GH (growth hormone). The main functions of IGF-1 include regulating the activity of GH and stimulating cell proliferation, including tenocyte formation [114, 116–118]. GH binds to IGF-1 binding proteins synthesized by the liver, mainly regulating the activity of IGF-1 [118–120]. Due to the increased release of GH from the pituitary gland, regular exercise results in a sustained increase in blood GH levels and IGF-1 levels, accompanied by increased IGF-1 mRNA expression in tendons [116]. However, the causal relationship between the upregulation of circulating GH/IGF-I and the stimulating effects of collagen synthesis in tendon healing remains to be elucidated [116]. Notably, GH is not seen to stimulate tendon healing in some studies using animal models [121, 122]. Crucially, the stimulating influence of IGF-I suggests that it can only play a local role, which cannot spread into a systemic effect. Similarly, studies have shown that load-induced IGF-I expression in skeletal muscle is unrelated to pituitary GH release [123, 124].

### 2.2. Transcription Factors and Relative Specific Proteins

In addition to the lack of tendon blood supply mentioned above, a common problem with tendon repair is that these regenerated tendons have poor histological characteristics and mechanical properties compared to normal tissue. Thus, the main problem of tendon differentiation is to find suitable conditions for stem cell differentiation. Transcription factors like Scx, early growth response 1 (EGR1), and Mohawk may offer promising solutions to these problems [125].

Scx plays a role as a transcription factor in tendon repair and regeneration. TGF-*β* signaling induces Scx expression in progenitor cells [126]. Normally, regeneration of damaged tendons requires that progenitor cells from the tissue surrounding the damaged area collect and bridge the defect. However, in Scx-deficient animals, progenitor cells move to the wound location but fail to correct for the deficit because ECM formation is impaired [126]. One study showed that Scx has an effect on human tendons during mechanical stimulation. After high physiological loading of the plantar tendon, the repair effect of Scx negative mice was inferior to that of the control group. Additionally, Scx-deficient mesenchymal stem cells have been reported to express fewer tendon-related genes than control cells [127]. Similarly, the ability of CD146+ pericytes to differentiate into tenocytes decreased in Scx-negative mice [128].

Guerquin et al. [129] first demonstrated the importance of early growth response 1(EGR1) in tendon regeneration by finding that forced EGR1 expression converts MSC into tendon differentiation by activating Scx, Col1a1, Col1a2, and other tendon-related collagen and molecules. Like Scx, the tendency of EGR1-MSC to differentiate into osteogenic or adipogenic cell lines is also hindered [129]. In addition, EGR1 has been proven to promote fibrin-based mesenchymal stem cell-engineered tendon formation in vitro. These EGR1-engineered tendons had larger diameters and higher levels of Scx, Col1a1, and Col1a2 expression in vitro compared to the control group and promoted better repair after implantation into a rat Achilles tendon injury model. Therefore, EGR1 has good potential in tendon repair and regeneration [129].

## 3. Mechanical Stimulation in Tendon Healing

To explore how mechanical stimulation affects tendon healing, extensive studies have been conducted in animal models. Although stretching disrupts tendon healing at the early stage of the inflammatory phase, the injured tendon can move in a controlled manner after the inflammatory period (about 7 days after injury). Moreover, the maximum strength and deviation characteristics of the healed tendon were improved [130, 131]. Early activity of the flexor tendon following injury also contributes to recovering sliding ability in people, improving mechanical properties, and stimulating the injured tendon to return to a normal shape [132].

By stimulating tenoblast activity (e.g., fibroblast proliferation, collagen synthesis, and rearrangement), mechanical stimulation through controlled mobilization promotes tendon repair and remodeling, resulting in increased tendon diameter and tensile strength and less adhesion than fixed healing tendon [133, 134]. To stimulate tendon healing, this process is accompanied by fibroblast proliferation and collagen rearrangement [135]. Compared with the control group, artificial fixation after tendon injury may result in poor tendon healing, mainly reflected in its reduced tensile strength and strain adjustment at the fracture site [136]. Additionally, immobilization reduced the moisture content in the tendon, and the content of proteoglycan also decreased [137]. Nevertheless, the positive effect of mechanical loading on healing depends on the type of tendon and the location of its damage due to the improved effect of cast fixation [138]. Therefore, the effect of mechanical loads on tendon healing varies according to type and area [139]. Differences in results may be due to variations in the mechanical requirements for different tendon regions and for different tendon types [2].

Tenoblasts can mediate the effects of mechanical stimulation through gene, protein, and cellular adjustments. Tendons respond to movement and speed up the healing process through these mechanical force transduction mechanisms. Mechanical loading promotes ECM production by stimulating the release of growth factors through the mechanisms described above. In addition, ECM turnover can also be adjusted by MMP regulation [140, 141].

Mechanical forces applied to the cell surface also cause effects in cytoskeletal structure that initiate complex signal transduction cascades within cells by activating integrins and stimulating G-protein-coupled receptors, receptor tyrosine kinases, and mitogen-activated protein kinases [142]. While the mechanical factors that play a significant role in the biology of tendon healing are known, the process by which mechanical stimuli are converted into biochemical reactions is not well understood. Changes in the ECM, cytoskeleton, and related gene transcription can be caused by mechanical factors acting on cells [143]. The role of mechanical stimulation in molecular and cellular responses in tendon development, homeostasis, aging, and healing remains to be explored. According to previous studies, two signaling pathways related to mechanical factors, TGF-*β*–SMad2/3 and FGF-ERK/MAPK, play a role in tendon healing. However, thus far, there is no conclusive evidence to prove whether there is an exact relationship between mechanical load, tendon healing-related genes, related growth factors, and tendon collagen synthesis [143]. In addition, mechanical force can activate the TGF-*β* signaling pathway to stimulate the expression of transcription factor Scx in rat models. This signaling pathway is mediated by SMAD2/3 [40]. Enhanced phosphorylation of ERK/MAPK was observed in vitro tendon tensile tests [144]. Moreover, transcription factors EGR1 and EGR2 encoded by mechanically sensitive genes are reported to be involved in tendon development in chickens and mice [145, 146]. Noticeably, after 15 minutes of mechanical loading, elevated levels of EGR1 and EGR2 gene expression were reported in injured rat's Achilles tendon [147, 148]. Therefore, it can be inferred that the role of mechanical forces in tendon healing and development is likely mediated by certain biological factors. One possibility is that EGR1 and EGR2 transcription factors are involved in tendon differentiation. Furthermore, a study in adult mice reported that EGR1 directly acts on the regulatory areas of TGF-*β*2 gene to take control of TGF-*β*2 transcription [129]. Based on these observations, it can be hypothesized that mechanical force can drive the generation of growth factors related to tendon differentiation and ultimately promote tendon differentiation by inducing transcription of molecular sensors. We suggest that similar mechanical transduction processes drive the proliferation and differentiation of tenocytes during the development, stabilization, and repair of tenocytes.

## 4. Prospects of Epigenetic Regulation in Tendon Healing

Humans and other mammals undergo epigenetic regulation of gene expression. Epigenetic mechanisms associated with molecular pathology and disease management approaches are sufficiently well understood for certain diseases such as cancer, cardiovascular disease, diabetes, and neurological disorders. Inflammation is a well-established epigenetic regulatory biological process, and inflammation-associated genes have been described in detail. However, there exists insufficient evidence supporting the role of epigenetic regulation of gene expression in tendon tissue healing. Inflammation is a common process of all tendinopathies associated with rotator cuff injury. Traditional treatment strategies have largely focused on dealing with pain and inflammation. However, since persistent disruption of ECM increases the risk of tendon injury recurrence, the possibility of epigenetic regulation of inflammatory genes and tendon repair-related gene expression cannot be ruled out [149].

Till date, about 2600 mature microRNAs (miRNAs) have been discovered in humans, and they are reported to be involved in the regulation of more than 60% of the encoding genes [150]. A unique feature of miRNAs compared to short interfering RNAs (siRNAs) is that they usually bind to target mRNAs in a partially complementary manner, meaning that a single miRNA can regulate hundreds of target genes. Consequently, changes in miRNA expression can profoundly affect biological processes such as cell proliferation, migration, differentiation, and apoptosis. Although miRNA sometimes plays an enhancer role, [150, 151] it is primarily a gene-silencing agent. Crucially, miRNA expression is negatively correlated with the regenerative potential of damaged tissues. Fetal mice displaying skin healing patterns at different stages of development demonstrates the regulatory role of miRNA in tissue regeneration [152]. Compared with late scar formation, early embryonic skin scar healing is characterized by an overall inhibition of miRNA function [152]. In addition, downregulation of miRNAs leads to cell multiplication and tissue regeneration in liver transplant patients [153]. These findings suggest that selective inhibition of miRNA functions in specific damaged tissues may potentially relieve silencing of tissue repair and regeneration genes and may restore tissue regeneration potential [154]. miRNA participates in maintaining the homeostasis of the internal environment of cells. Involved in the development of tendinopathy, miRNA induces mRNA degradation and inhibits gene translation [144, 155–159]. A series of previous studies from our laboratory have established that miRNA regulates important factors in tendon healing, including bone morphogenetic protein- (BMP-) 2, BMP-7, interleukin- (IL-) 6, and collagen types I and III [53, 160]. In the differentiation and guidance of TDSCs, different miRNAs promote or inhibit TDSCs by targeting different genes [161–163]. In addition, miRNA also contributes to reducing adhesion of healing tendon and promoting tendon remodeling [164]. Treatments based on miRNA or miRNA inhibitors have considerable therapeutic potential in a variety of diseases, including cancer. Although miRNAs targeting multiple mRNAs involved in either same or different pathways offer a potential therapeutic choice, choosing which pathway to target requires careful evaluation, and consideration should be given to nonspecific targets [156].

In recent years, it was discovered that long noncoding RNAs (lncRNAs) widely transcribed from mammalian genomes have several biological functions [165, 166]. For example, lncRNAs are found to be crucial regulators of various biological activities and disease progression [167, 168]. Further, recent data suggest that lncRNAs regulate cellular differentiation and tissue regeneration [169–172]. lncRNA H19 is an imprinted gene [173] which was discovered more than 20 years ago. By now, it is well established that lncRNA H19 is located on human chromosome 11; however, its function is not fully understood. H19 is highly expressed in embryonic tissue that originates from the endoderm and mesoderm. However, after birth, it is uninhibited only in the skeletal muscle [174, 175]. Further, H19 is reported to be a new tendon differentiation activator of TDSCs. Furthermore, enhanced expression of H19 accelerated the differentiation of tendons induced by TGF-*β*1 in vitro and facilitated tendon proliferation in vivo [162]. In addition, lncRNA can also synergistically interact with miRNAs, and lncRNA X-inactive specific transcript (XIST) prevents the adhesion of healing tendons via miR-26a-5p/COX2 pathway and promotes tendon healing [176].

In addition to miRNA and lncRNA, the role of other epigenetic regulation mechanisms in tendon healing is currently being explored. It is suggested that understanding the DNA methylation patterns and histone modification mechanisms of tendon-specific inflammation genes will further our understanding of tendon pathology and may even lead to the development of novel treatment/management strategies [156].

## 5. Conclusion

As a complex human tissue, tendons have a unique structure and perform a specific mechanical function. Tendon repair after an injury is a complex process that may involve subsequent complications including adhesion formation. Multiple factors are involved in tendon healing such as mechanical and biochemical factors and epigenetic regulation. From a holistic point of view, the tendon-healing process should be stimulated by mechanical action from gene expression to a series of biochemical factors and finally the formation of proteins to perform biological functions.

While we have a good understanding of the framework of tendon healing, there are still several mechanisms including a few pathways mentioned above and certain mechanisms of epigenetic regulation that are still being explored. Noticeably, the latest research indicates that only epigenetically regulated miRNAs play a decisive role in promoting mechanical strength during tendon healing while simultaneously reducing the formation of adhesions. The role of VEGF remains unclear. Adhesion is the result of incoordination between intrinsic and extrinsic healing. Our review clearly points out that biochemical factors only promote healing by alleviating adhesions. Therefore, we advocate that to better understand the tendon-healing process, upstream epigenetic regulatory mechanisms should be identified by future studies. Hence, the exploration of epigenetic regulation is a promising direction in the field of tendon healing.

## Figures and Tables

**Table 1:** Influencing factors in tendon healing process.

Factor		Effects on tendon healing	
Mechanical factors	Overloading	Helps the middle tendon repair but inhibits healing at the enthesis.	[139, 177–179]
	Underloading	Inhibits the middle tendon repair but helps healing at the enthesis.	[177–180]

Transcription factors	Scx, EGR1, and Mohawk	Promote tendon repair and regeneration and provide conditions conducive to cell differentiation.	[2]

Signaling	TGF-*β*	Involved in initial inflammatory responses, collagen synthesis, and angiogenesis.	[79]
	VEGF	Promotes angiogenesis and fibroblast proliferation and activates the synthesis of other growth factors.	[79]
	FGF	Involved in inflammation, angiogenesis, cell proliferation, and collagen synthesis.	[79]
	PDGF	Promotes angiogenesis, ECM synthesis, tenoblast migration, and differentiation.	[79]
	IGF	Promotes collagen synthesis in fibroblasts and ECM synthesis.	[79]
	BMP	Improves molecular, organizational, and mechanical properties of healing tendon.	[181–184]

Epigenetic regulation	miRNA and lncRNA	Guide tendon stem cell differentiation and tissue regeneration, promote tendon healing, and reduce adhesion.	[161–163]

EGR1: early growth response 1; Scx: scleraxis; TGF-*β*: transformational growth factor-beta; VEGF: vascular endothelial growth factor; FGF: fibroblast growth factor; PDGF: platelet-derived growth factor; ECM: extracellular matrix; IGF: insulin-like growth factor; BMP: bone morphogenetic protein.

## References

[1] Benjamin M., Ralphs J. R. (1997). Tendons and ligaments--an overview. *Histology and Histopathology*.

[2] Nourissat G., Berenbaum F., Duprez D. (2015). Tendon injury: from biology to tendon repair. *Nature Reviews Rheumatology*.

[3] Asahara H., Inui M., Lotz M. K. (2017). Tendons and ligaments: connecting developmental biology to musculoskeletal disease pathogenesis. *Journal of Bone and Mineral Research*.

[4] Apostolakos J., Durant T. J., Dwyer C. R. (2014). The enthesis: a review of the tendon-to-bone insertion. *Muscles, Ligaments and Tendons Journal*.

[5] Dakin S. G., Martinez F. O., Yapp C. (2015). Inflammation activation and resolution in human tendon disease. *Science Translational Medicine*.

[6] Gwilym S. E., Watkins B., Cooper C. D. (2009). Genetic influences in the progression of tears of the rotator cuff. *The Journal of Bone and Joint Surgery. British Volume*.

[7] Dudhia J., Scott C. M., Draper E. R., Heinegård D., Pitsillides A. A., Smith R. K. (2007). Aging enhances a mechanically-induced reduction in tendon strength by an active process involving matrix metalloproteinase activity. *Aging Cell*.

[8] de Jong J. P., Nguyen J. T., Sonnema A. J., Nguyen E. C., Amadio P. C., Moran S. L. (2014). The incidence of acute traumatic tendon injuries in the hand and wrist: a 10-year population-based study. *Clinics in Orthopedic Surgery*.

[9] Li Z. J., Yang Q. Q., Zhou Y. L. (2021). Basic research on tendon repair: strategies, Evaluation, and Development. *Frontiers in medicine*.

[10] Kaux J. F., Forthomme B., Goff C. L., Crielaard J. M., Croisier J. L. (2011). Current opinions on tendinopathy. *Journal of Sports Science & Medicine*.

[11] Snedeker J. G., Foolen J. (2017). Tendon injury and repair - a perspective on the basic mechanisms of tendon disease and future clinical therapy. *Acta Biomaterialia*.

[12] Tempfer H., Traweger A. (2015). Tendon vasculature in health and disease. *Frontiers in Physiology*.

[13] Evrova O., Buschmann J. (2017). In vitro and in vivo effects of PDGF-BB delivery strategies on tendon healing: a review. *European Cells & Materials*.

[14] Meier Bürgisser G., Calcagni M., Bachmann E. (2016). Rabbit Achilles tendon full transection model - wound healing, adhesion formation and biomechanics at 3, 6 and 12 weeks post-surgery. *Biology Open*.

[15] Galatz L. M., Gerstenfeld L., Heber-Katz E., Rodeo S. A. (2015). Tendon regeneration and scar formation: the concept of scarless healing. *Journal of Orthopaedic Research : Official Publication of the Orthopaedic Research Society*.

[16] Wu W., Cheng R., das Neves J. (2017). Advances in biomaterials for preventing tissue adhesion. *Journal of Controlled Release : Official Journal of the Controlled Release Society*.

[17] Korntner S., Zeugolis D. I. (2019). Wound healing and fibrosis - state of play. *Advanced Drug Delivery Reviews*.

[18] Zhang Q., Yang Y., Yildirimer L., Xu T., Zhao X. (2021). Advanced technology-driven therapeutic interventions for prevention of tendon adhesion: design, intrinsic and extrinsic factor considerations. *Acta Biomaterialia*.

[19] Legrand A., Kaufman Y., Long C., Fox P. M. (2017). Molecular biology of flexor tendon healing in relation to reduction of tendon adhesions. *The Journal of Hand Surgery*.

[20] Zhao X., Jiang S., Liu S. (2015). Optimization of intrinsic and extrinsic tendon healing through controllable water-soluble mitomycin-C release from electrospun fibers by mediating adhesion-related gene expression. *Biomaterials*.

[21] Lim W. L., Liau L. L., Ng M. H., Chowdhury S. R., Law J. X. (2019). Current progress in tendon and ligament tissue engineering. *Tissue Engineering and Regenerative Medicine*.

[22] Shen H., Jayaram R., Yoneda S. (2018). The effect of adipose-derived stem cell sheets and CTGF on early flexor tendon healing in a canine model. *Scientific Reports*.

[23] Walia B., Huang A. H. (2019). Tendon stem progenitor cells: understanding the biology to inform therapeutic strategies for tendon repair. *Journal of Orthopaedic Research : Official Publication of the Orthopaedic Research Society*.

[24] Leadbetter W. B. (1992). Cell-matrix response in tendon injury. *Clinics in Sports Medicine*.

[25] Hope M., Saxby T. S. (2007). Tendon healing. *Foot and Ankle Clinics*.

[26] Bedi A., Kovacevic D., Hettrich C. (2010). The effect of matrix metalloproteinase inhibition on tendon-to-bone healing in a rotator cuff repair model. *Journal of Shoulder and Elbow Surgery*.

[27] Sun H. B., Andarawis-Puri N., Li Y. (2010). Cycle-dependent matrix remodeling gene expression response in fatigue-loaded rat patellar tendons. *Journal of Orthopaedic Research : Official Publication of the Orthopaedic Research Society*.

[28] Dalton S., Cawston T. E., Riley G. P., Bayley I. J., Hazleman B. L. (1995). Human shoulder tendon biopsy samples in organ culture produce procollagenase and tissue inhibitor of metalloproteinases. *Annals of the Rheumatic Diseases*.

[29] Oshiro W., Lou J., Xing X., Tu Y., Manske P. R. (2003). Flexor tendon healing in the rat: a histologic and gene expression study. *The Journal of Hand Surgery*.

[30] Wu G., Sun B., Zhao C. (2021). Three-dimensional tendon scaffold loaded with TGF-*β*1 gene silencing plasmid prevents tendon adhesion and promotes tendon repair. *ACS Biomaterials Science & Engineering*.

[31] Aschner Y., Downey G. P. (2016). Transforming growth factor-*β*: master regulator of the respiratory system in health and disease. *American Journal of Respiratory Cell and Molecular Biology*.

[32] Thomopoulos S., Parks W. C., Rifkin D. B., Derwin K. A. (2015). Mechanisms of tendon injury and repair. *Journal of Orthopaedic Research : Official Publication of the Orthopaedic Research Society*.

[33] Katzel E. B., Wolenski M., Loiselle A. E. (2011). Impact of Smad3 loss of function on scarring and adhesion formation during tendon healing. *Journal of Orthopaedic Research : Official Publication of the Orthopaedic Research Society*.

[34] Chang J., Most D., Stelnicki E. (1997). Gene expression of transforming growth factor beta-1 in rabbit zone II flexor tendon wound healing: evidence for dual mechanisms of repair. *Plastic and Reconstructive Surgery*.

[35] Pryce B. A., Watson S. S., Murchison N. D., Staverosky J. A., Dünker N., Schweitzer R. (2009). Recruitment and maintenance of tendon progenitors by TGFbeta signaling are essential for tendon formation. *Development (Cambridge, England)*.

[36] Brown J. P., Finley V. G., Kuo C. K. (2014). Embryonic mechanical and soluble cues regulate tendon progenitor cell gene expression as a function of developmental stage and anatomical origin. *Journal of Biomechanics*.

[37] Berthet E., Chen C., Butcher K., Schneider R. A., Alliston T., Amirtharajah M. (2013). Smad3 binds scleraxis and Mohawk and regulates tendon matrix organization. *Journal of Orthopaedic Research : Official Publication of the Orthopaedic Research Society*.

[38] Massagué J. (2012). TGF*β* signalling in context. *Nature Reviews Molecular Cell Biology*.

[39] Havis E., Bonnin M. A., Olivera-Martinez I. (2014). Transcriptomic analysis of mouse limb tendon cells during development. *Development (Cambridge, England)*.

[40] Jiang K., Chun G., Wang Z., Du Q., Wang A., Xiong Y. (2016). Effect of transforming growth factor-*β*3 on the expression of Smad3 and Smad7 in tenocytes. *Molecular Medicine Reports*.

[41] Maeda T., Sakabe T., Sunaga A. (2011). Conversion of mechanical force into TGF-*β*-mediated biochemical signals. *Current Biology*.

[42] Wang Y., Zhou Z., Liu Y., Wang Z., Kang Y. (2021). Inhibition of Smad3 promotes the healing of rotator cuff injury in a rat model. *Journal of Orthopaedic Research : Official Publication of the Orthopaedic Research Society*.

[43] Wu Y. F., Mao W. F., Zhou Y. L., Wang X. T., Liu P. Y., Tang J. B. (2016). Adeno-associated virus-2-mediated TGF-*β*1 microRNA transfection inhibits adhesion formation after digital flexor tendon injury. *Gene Therapy*.

[44] Tsubone T., Moran S. L., Amadio P. C., Zhao C., An K. N. (2004). Expression of growth factors in canine flexor tendon after laceration in vivo. *Annals of Plastic Surgery*.

[45] Chan K. M., Fu S. C., Wong Y. P., Hui W. C., Cheuk Y. C., Wong M. W. (2008). Expression of transforming growth factor beta isoforms and their roles in tendon healing. *Wound Repair and Regeneration*.

[46] Farhat Y. M., Al-Maliki A. A., Chen T. (2012). Gene expression analysis of the pleiotropic effects of TGF-*β*1 in an in vitro model of flexor tendon healing. *PLoS One*.

[47] Hou Y., Mao Z., Wei X. (2009). Effects of transforming growth factor-*β*1 and vascular endothelial growth factor 165 gene transfer on Achilles tendon healing. *Matrix Biology*.

[48] Kapacee Z., Yeung C. Y., Lu Y., Crabtree D., Holmes D. F., Kadler K. E. (2010). Synthesis of embryonic tendon-like tissue by human marrow stromal/mesenchymal stem cells requires a three-dimensional environment and transforming growth factor *β*3. *Matrix Biology*.

[49] Manning C. N., Kim H. M., Sakiyama-Elbert S., Galatz L. M., Havlioglu N., Thomopoulos S. (2011). Sustained delivery of transforming growth factor beta three enhances tendon-to-bone healing in a rat model. *Journal of Orthopaedic Research : Official Publication of the Orthopaedic Research Society*.

[50] Kaji D. A., Tan Z., Johnson G. L. (2020). Cellular plasticity in musculoskeletal Development, Regeneration, and Disease. *Regeneration, and Disease, Journal of orthopaedic research*.

[51] Petersen W., Pufe T., Unterhauser F., Zantop T., Mentlein R., Weiler A. (2003). The splice variants 120 and 164 of the angiogenic peptide vascular endothelial cell growth factor (VEGF) are expressed during Achilles tendon healing. *Archives of Orthopaedic and Trauma Surgery*.

[52] Apte R. S., Chen D. S., Ferrara N. (2019). VEGF in signaling and disease: beyond discovery and development. *Cell*.

[53] Hall K., Ran S. (2010). Regulation of tumor angiogenesis by the local environment. *Frontiers in Bioscience (Landmark edition)*.

[54] Nakamura K., Kitaoka K., Tomita K. (2008). Effect of eccentric exercise on the healing process of injured patellar tendon in rats. *Journal of Orthopaedic Science : Official Journal of the Japanese Orthopaedic Association*.

[55] Korntner S., Lehner C., Gehwolf R. (2019). Limiting angiogenesis to modulate scar formation. *Advanced Drug Delivery Reviews*.

[56] Peach C. J., Mignone V. W., Arruda M. A. (2018). Molecular pharmacology of VEGF-A isoforms: binding and signalling at VEGFR2. *International Journal of Molecular Sciences*.

[57] Wu F., Nerlich M., Docheva D. (2017). Tendon injuries: basic science and new repair proposals. *EFORT Open Reviews*.

[58] Molloy T., Wang Y., Murrell G. (2003). The roles of growth factors in tendon and ligament Healing. *Sports Medicine (Auckland, N.Z.)*.

[59] Parsons-Wingerter P., Chandrasekharan U. M., McKay T. L. (2006). A VEGF_165_-induced phenotypic switch from increased vessel density to increased vessel diameter and increased endothelial NOS activity. *Microvascular Research*.

[60] Liu X., Zhang R., Zhu B. (2021). Effects of leukocyte- and platelet-rich plasma on tendon disorders based on in vitro and in vivo studies (review). *Experimental and Therapeutic Medicine*.

[61] Zhang F., Liu H., Stile F. (2003). Effect of vascular endothelial growth factor on rat Achilles tendon healing. *Plastic and Reconstructive Surgery*.

[62] Alexander S. P., Fabbro D., Kelly E. (2015). The concise guide to PHARMACOLOGY 2015/16: catalytic receptors. *British Journal of Pharmacology*.

[63] Rahim M., El Khoury L. Y., Raleigh S. M. (2016). Human genetic variation, Sport and Exercise Medicine, and Achilles Tendinopathy: Role for Angiogenesis-Associated Genes. *Omics : A Journal of Integrative Biology*.

[64] Halper J. (2014). Advances in the use of growth factors for treatment of disorders of soft tissues. *Advances in Experimental Medicine and Biology*.

[65] Li W., Kohara H., Uchida Y. (2013). Peripheral nerve-derived CXCL12 and VEGF-A regulate the patterning of arterial vessel branching in developing limb skin. *Developmental Cell*.

[66] Pufe T., Petersen W. J., Mentlein R., Tillmann B. N. (2005). The role of vasculature and angiogenesis for the pathogenesis of degenerative tendons disease. *Scandinavian Journal of Medicine & Science in Sports*.

[67] Liu X., Zhu B., Li Y. (2021). The role of vascular endothelial growth factor in tendon healing. *Frontiers in Physiology*.

[68] Lakemeier S., Reichelt J. J., Patzer T., Fuchs-Winkelmann S., Paletta J. R., Schofer M. D. (2010). The association between retraction of the torn rotator cuff and increasing expression of hypoxia inducible factor 1*α* and vascular endothelial growth factor expression: an immunohistological study. *BMC Musculoskeletal Disorders*.

[69] Li Z., Meyers C. A., Chang L. (2019). Fracture repair requires TrkA signaling by skeletal sensory nerves. *The Journal of Clinical Investigation*.

[70] Lee S., Hwang C., Marini S. (2021). NGF-TrkA signaling dictates neural ingrowth and aberrant osteochondral differentiation after soft tissue trauma. *Nature Communications*.

[71] Tempfer H., Wagner A., Gehwolf R. (2009). Perivascular cells of the supraspinatus tendon express both tendon- and stem cell-related markers. *Histochemistry and Cell Biology*.

[72] Cui J., Chen Z., Wu W. (2019). Expression of TGF-*β*1 and VEGF in patients with Achilles tendon rupture and the clinical efficacy. *Experimental and Therapeutic Medicine*.

[73] Yoshikawa T., Tohyama H., Enomoto H., Matsumoto H., Toyama Y., Yasuda K. (2006). Expression of vascular endothelial growth factor and angiogenesis in patellar tendon grafts in the early phase after anterior cruciate ligament reconstruction. *Knee Surgery, Sports Traumatology, Arthroscopy*.

[74] Bosch G., Moleman M., Barneveld A., van Weeren P. R., van Schie H. T. (2011). The effect of platelet-rich plasma on the neovascularization of surgically created equine superficial digital flexor tendon lesions. *Scandinavian Journal of Medicine & Science in Sports*.

[75] Järvinen T. A. (2020). Neovascularisation in tendinopathy: from eradication to stabilisation?. *British Journal of Sports Medicine*.

[76] Sahin H., Tholema N., Petersen W., Raschke M. J., Stange R. (2012). Impaired biomechanical properties correlate with neoangiogenesis as well as VEGF and MMP-3 expression during rat patellar tendon healing. *Journal of Orthopaedic Research*.

[77] Zhang J., Liu Z., Li Y. (2020). FGF2: a key regulator augmenting tendon-to-bone healing and cartilage repair. *Regenerative Medicine*.

[78] Platonova N., Miquel G., Chiu L. Y. (2014). Dimerization capacities of FGF2 purified with or without heparin-affinity chromatography. *PLoS One*.

[79] Titan A. L., Foster D. S., Chang J., Longaker M. T. (2019). Flexor tendon: development, healing, adhesion formation, and contributing growth factors. *Plastic and Reconstructive Surgery*.

[80] Tang J. B., Wu Y. F., Cao Y. (2016). Basic FGF or VEGF gene therapy corrects insufficiency in the intrinsic healing capacity of tendons. *Scientific Reports*.

[81] Thomopoulos S., Kim H. M., Das R. (2010). The effects of exogenous basic fibroblast growth factor on intrasynovial flexor tendon healing in a canine model. *The Journal of Bone and Joint Surgery. American*.

[82] Kobayashi M., Itoi E., Minagawa H. (2006). Expression of growth factors in the early phase of supraspinatus tendon healing in rabbits. *Journal of Shoulder and Elbow Surgery*.

[83] Hankemeier S., Keus M., Zeichen J. (2005). Modulation of proliferation and differentiation of human bone marrow stromal cells by fibroblast growth factor 2: potential implications for tissue engineering of tendons and ligaments. *Tissue Engineering*.

[84] Zhang C., Li Q., Deng S. (2016). bFGF- and CaPP-loaded fibrin clots enhance the bioactivity of the tendon-bone interface to augment healing. *The American Journal of Sports Medicine*.

[85] Raica M., Cimpean A. M. (2010). Platelet-derived growth factor (PDGF)/PDGF receptors (PDGFR) axis as target for antitumor and antiangiogenic therapy. *Pharmaceuticals (Basel, Switzerland)*.

[86] Sugg K. B., Markworth J. F., Disser N. P. (2018). Postnatal tendon growth and remodeling require platelet-derived growth factor receptor signaling. *American Journal of Physiology. Cell Physiology*.

[87] Scherping S. C., Schmidt C. C., Georgescu H. I., Kwoh C. K., Evans C. H., Woo S. L. (1997). Effect of growth factors on the proliferation of ligament fibroblasts from skeletally mature rabbits. *Connective Tissue Research*.

[88] Hee C. K., Dines J. S., Dines D. M. (2011). Augmentation of a rotator cuff suture repair using rhPDGF-BB and a type I bovine collagen matrix in an ovine model. *The American Journal of Sports Medicine*.

[89] Suwalski A., Dabboue H., Delalande A. (2010). Accelerated Achilles tendon healing by PDGF gene delivery with mesoporous silica nanoparticles. *Biomaterials*.

[90] Hee C. K., Dines J. S., Solchaga L. A., Shah V. R., Hollinger J. O. (2012). Regenerative tendon and ligament healing: opportunities with recombinant human platelet-derived growth factor BB-homodimer. *Tissue engineering. Part B, Reviews*.

[91] Thomopoulos S., Zaegel M., Das R. (2007). PDGF-BB released in tendon repair using a novel delivery system promotes cell proliferation and collagen remodeling. *Journal of Orthopaedic Research : Official Publication of the Orthopaedic Research Society*.

[92] Anitua E., Sanchez M., Nurden A. T. (2007). Reciprocal actions of platelet-secreted TGF-beta1 on the production of VEGF and HGF by human tendon cells. *Plastic and Reconstructive Surgery*.

[93] Urist M. R. (1965). Bone: formation by autoinduction. *Science (New York, N.Y.)*.

[94] Miyazono K., Maeda S., Imamura T. (2005). BMP receptor signaling: transcriptional targets, regulation of signals, and signaling cross-talk. *Cytokine & Growth Factor Reviews*.

[95] Guo J., Wu G. (2012). The signaling and functions of heterodimeric bone morphogenetic proteins. *Cytokine & Growth Factor Reviews*.

[96] Aspenberg P., Forslund C. (1999). Enhanced tendon healing with GDF 5 and 6. *Acta Orthopaedica Scandinavica*.

[97] D'Souza D., Patel K. (1999). Involvement of long- and short-range signalling during early tendon development. *Anatomy and Embryology*.

[98] Dudakovic A., Samsonraj R. M., Paradise C. R. (2020). Inhibition of the epigenetic suppressor EZH2 primes osteogenic differentiation mediated by BMP2. *The Journal of Biological Chemistry*.

[99] Bolt P., Clerk A. N., Luu H. H. (2007). BMP-14 gene therapy increases tendon tensile strength in a rat model of Achilles tendon injury. *The Journal of Bone and Joint Surgery. America*.

[100] Dines J. S., Weber L., Razzano P. (2007). The effect of growth differentiation factor-5-coated sutures on tendon repair in a rat model. *Journal of Shoulder and Elbow Surgery*.

[101] Forslund C., Rueger D., Aspenberg P. (2003). A comparative dose-response study of cartilage-derived morphogenetic protein (CDMP)-1, -2 and -3 for tendon healing in rats. *Journal of Orthopaedic Research : Official Publication of the Orthopaedic Research Society*.

[102] Tan S. L., Ahmad R. E., Ahmad T. S. (2012). Effect of growth differentiation factor 5 on the proliferation and tenogenic differentiation potential of human mesenchymal stem cells in vitro. *Cells, Tissues, Organs*.

[103] Helm G. A., Li J. Z., Alden T. D. (2001). A light and electron microscopic study of ectopic tendon and ligament formation induced by bone morphogenetic protein-13 adenoviral gene therapy. *Journal of Neurosurgery*.

[104] Wang Q. W., Chen Z. L., Piao Y. J. (2005). Mesenchymal stem cells differentiate into tenocytes by bone morphogenetic protein (BMP) 12 gene transfer. *Journal of Bioscience and Bioengineering*.

[105] Lou J., Tu Y., Burns M., Silva M. J., Manske P. (2001). BMP-12 gene transfer augmentation of lacerated tendon repair. *Journal of Orthopaedic Research : Official Publication of the Orthopaedic Research Society*.

[106] Violini S., Ramelli P., Pisani L. F., Gorni C., Mariani P. (2009). Horse bone marrow mesenchymal stem cells express embryo stem cell markers and show the ability for tenogenic differentiation by in vitro exposure to BMP-12. *BMC Cell Biology*.

[107] Kang Q., Song W. X., Luo Q. (2009). A comprehensive analysis of the dual roles of BMPs in regulating adipogenic and osteogenic differentiation of mesenchymal progenitor cells. *Stem Cells and Development*.

[108] Rui Y. F., Lui P. P., Ni M., Chan L. S., Lee Y. W., Chan K. M. (2011). Mechanical loading increased BMP-2 expression which promoted osteogenic differentiation of tendon-derived stem cells. *Journal of Orthopaedic Research : Official Publication of the Orthopaedic Research Society*.

[109] Rui Y. F., Lui P. P., Wong Y. M., Tan Q., Chan K. M. (2013). BMP-2 stimulated non-tenogenic differentiation and promoted proteoglycan deposition of tendon-derived stem cells (TDSCs) in vitro. *Journal of Orthopaedic Research : Official Publication of the Orthopaedic Research Society*.

[110] Kelley K. M., Schmidt K. E., Berg L. (2002). Comparative endocrinology of the insulin-like growth factor-binding protein. *The Journal of Endocrinology*.

[111] Heinemeier K. M., Mackey A. L., Doessing S. (2012). GH/IGF-I axis and matrix adaptation of the musculotendinous tissue to exercise in humans. *Scandinavian Journal of Medicine & Science in Sports*.

[112] Banes A. J., Tsuzaki M., Hu P. (1995). PDGF-BB, IGF-I and mechanical load stimulate DNA synthesis in avian tendon fibroblasts *in vitro*. *Journal of Biomechanics*.

[113] Hansen M., Boesen A., Holm L., Flyvbjerg A., Langberg H., Kjaer M. (2013). Local administration of insulin-like growth factor-I (IGF-I) stimulates tendon collagen synthesis in humans. *Scandinavian Journal of Medicine & Science in Sports*.

[114] Hernandez D. M., Kang J. H., Choudhury M. (2020). IPF pathogenesis is dependent upon TGF*β* induction of IGF-1. *FASEB Journal : Official Publication of the Federation of American Societies for Experimental Biology*.

[115] Ghahary A., Tredget E. E., Shen Q., Kilani R. T., Scott P. G., Houle Y. (2000). Mannose-6-phosphate/IGF-II receptors mediate the effects of IGF-1-induced latent transforming growth factor beta 1 on expression of type I collagen and collagenase in dermal fibroblasts. *Growth Factors (Chur, Switzerland)*.

[116] Ramos D. M., Abdulmalik S., Arul M. R. (2019). Insulin immobilized PCL-cellulose acetate micro-nanostructured fibrous scaffolds for tendon tissue engineering. *Polymers for Advanced Technologies*.

[117] Tsuzaki M., Brigman B. E., Yamamoto J. (2000). Insulin-like growth factor-I is expressed by avian flexor tendon cells. *Journal of Orthopaedic Research : Official Publication of the Orthopaedic Research Society*.

[118] Halper J. (2010). Growth factors as active participants in carcinogenesis: a perspective. *Veterinary Pathology*.

[119] Brown C. A., Halper J. (1990). Mitogenic effects of transforming growth factor type e on epithelial and fibroblastic cells--comparison with other growth factors. *Experimental Cell Research*.

[120] Juul A. (2003). Serum levels of insulin-like growth factor I and its binding proteins in health and disease. *Growth Hormone & IGF Research*.

[121] Provenzano P. P., Alejandro-Osorio A. L., Grorud K. W. (2007). Systemic administration of IGF-I enhances healing in collagenous extracellular matrices: evaluation of loaded and unloaded ligaments. *BMC Physiology*.

[122] Andersson T., Eliasson P., Aspenberg P. (2012). Growth hormone does not stimulate early healing in rat tendons. *International Journal of Sports Medicine*.

[123] Abate M., Guelfi M., Pantalone A. (2016). Therapeutic use of hormones on tendinopathies: a narrative review. *Muscles, Ligaments and Tendons Journal*.

[124] Yamaguchi A., Fujikawa T., Shimada S. (2006). Muscle IGF-I Ea, MGF, and myostatin mRNA expressions after compensatory overload in hypophysectomized rats. *Pflugers Archiv : European Journal of Physiology*.

[125] Liu H., Zhu S., Zhang C. (2014). Crucial transcription factors in tendon development and differentiation: their potential for tendon regeneration. *Cell and Tissue Research*.

[126] He P., Ruan D., Huang Z. (2022). Comparison of tendon development versus tendon healing and regeneration. *Frontiers in Cell and Developmental Biology*.

[127] Alberton P., Popov C., Prägert M. (2012). Conversion of human bone marrow-derived mesenchymal stem cells into tendon progenitor cells by ectopic expression of scleraxis. *Stem Cells and Development*.

[128] Gumucio J. P., Schonk M. M., Kharaz Y. A., Comerford E., Mendias C. L. (2020). Scleraxis is required for the growth of adult tendons in response to mechanical loading. *JCI Insight*.

[129] Guerquin M. J., Charvet B., Nourissat G. (2013). Transcription factor EGR1 directs tendon differentiation and promotes tendon repair. *The Journal of Clinical Investigation*.

[130] Gelberman R. H., Manske P. R., Akeson W. H., Woo S. L., Lundborg G., Amiel D. (1986). Flexor tendon repair. *Journal of Orthopaedic Research : Official Publication of the Orthopaedic Research Society*.

[131] Wada A., Kubota H., Miyanishi K., Hatanaka H., Miura H., Iwamoto Y. (2001). Comparison of postoperative early active mobilization and immobilization in vivo utilising a four-strand flexor tendon repair. *Journal of Hand Surgery (Edinburgh, Scotland)*.

[132] Thien T. B., Becker J. H., Theis J. C. (2004). Rehabilitation after surgery for flexor tendon injuries in the hand. *The Cochrane Database of Systematic Reviews*.

[133] Kannus P. (1997). Tendons--a source of major concern in competitive and recreational athletes. *Scandinavian Journal of Medicine & Science in Sports*.

[134] Wang J. H. (2006). Mechanobiology of tendon. *Journal of Biomechanics*.

[135] Davidson C. J., Ganion L. R., Gehlsen G. M., Verhoestra B., Roepke J. E., Sevier T. L. (1997). Rat tendon morphologic and functional changes resulting from soft tissue mobilization. *Medicine and Science in Sports and Exercise*.

[136] Yamamoto E., Hayashi K., Yamamoto N. (1999). Mechanical properties of collagen fascicles from stress-shielded patellar tendons in the rabbit. *Clinical Biomechanics (Bristol, Avon)*.

[137] Akeson W. H., Amiel D., Mechanic G. L., Woo S. L., Harwood F. L., Hamer M. L. (1977). Collagen cross-linking alterations in joint contractures: changes in the reducible cross-links in periarticular connective tissue collagen after nine weeks of immobilization. *Connective Tissue Research*.

[138] Gimbel J. A., Van Kleunen J. P., Williams G. R., Thomopoulos S., Soslowsky L. J. (2007). Long durations of immobilization in the rat result in enhanced mechanical properties of the healing supraspinatus tendon insertion site. *Journal of Biomechanical Engineering*.

[139] Killian M. L., Cavinatto L., Galatz L. M., Thomopoulos S. (2012). The role of mechanobiology in tendon healing. *Journal of Shoulder and Elbow Surgery*.

[140] Skutek M., van Griensven M., Zeichen J., Brauer N., Bosch U. (2001). Cyclic mechanical stretching modulates secretion pattern of growth factors in human tendon fibroblasts. *European Journal of Applied Physiology*.

[141] Tsuzaki M., Bynum D., Almekinders L., Yang X., Faber J., Banes A. J. (2003). ATP modulates load-inducible IL-1beta, COX 2, and MMP-3 gene expression in human tendon cells. *Journal of Cellular Biochemistry*.

[142] Wang N., Ingber D. E. (1995). Probing transmembrane mechanical coupling and cytomechanics using magnetic twisting cytometry. *Biochimie et Biologie Cellulaire*.

[143] Humphrey J. D., Dufresne E. R., Schwartz M. A. (2014). Mechanotransduction and extracellular matrix homeostasis. *Nature Reviews. Molecular Cell Biology*.

[144] Thankam F. G., Boosani C. S., Dilisio M. F., Dietz N. E., Agrawal D. K. (2016). MicroRNAs associated with shoulder tendon matrisome disorganization in glenohumeral arthritis. *PLoS One*.

[145] Pagel J. I., Deindl E. (2011). Early growth response 1--a transcription factor in the crossfire of signal transduction cascades. *Indian Journal of Biochemistry & Biophysics*.

[146] Lejard V., Blais F., Guerquin M. J. (2011). EGR1 and EGR2 Involvement in Vertebrate Tendon Differentiation. *The Journal of Biological Chemistry*.

[147] Hammerman M., Aspenberg P., Eliasson P. (2014). Microtrauma stimulates rat Achilles tendon healing via an early gene expression pattern similar to mechanical loading. *Journal of Applied Physiology (Bethesda, Md. : 1985)*.

[148] Eliasson P., Andersson T., Hammerman M., Aspenberg P. (2013). Primary gene response to mechanical loading in healing rat Achilles tendons. *Journal of Applied Physiology (Bethesda, Md. : 1985)*.

[149] Thankam F. G., Boosani C. S., Dilisio M. F., Agrawal D. K. (2019). Epigenetic mechanisms and implications in tendon inflammation (review). *International Journal of Molecular Medicine*.

[150] Fineberg S. K., Kosik K. S., Davidson B. L. (2009). MicroRNAs potentiate neural development. *Neuron*.

[151] Kwok G. T. Y., Zhao J. T., Glover A. R. (2019). MicroRNA-431 as a chemosensitizer and potentiator of drug activity in adrenocortical carcinoma. *The Oncologist*.

[152] Cheng J., Yu H., Deng S., Shen G. (2010). MicroRNA profiling in mid- and late-gestational fetal skin: implication for scarless wound healing. *The Tohoku Journal of Experimental Medicine*.

[153] Salehi S., Brereton H. C., Arno M. J. (2013). Human liver regeneration is characterized by the coordinated expression of distinct microRNA governing cell cycle fate. *American Journal of Transplantation : Official Journal of the American Society of Transplantation and the American Society of Transplant Surgeons*.

[154] Schwartzfarb E., Kirsner R. S. (2012). Understanding scarring: scarless fetal wound healing as a model. *The Journal of Investigative Dermatology*.

[155] Treiber T., Treiber N., Meister G. (2018). Author correction: regulation of microRNA biogenesis and its crosstalk with other cellular pathways. *Nature Reviews. Molecular Cell Biology*.

[156] Thankam F. G., Boosani C. S., Dilisio M. F., Agrawal D. K. (2018). MicroRNAs associated with inflammation in shoulder tendinopathy and glenohumeral arthritis. *Molecular and Cellular Biochemistry*.

[157] Plachel F., Heuberer P., Gehwolf R. (2020). MicroRNA profiling reveals distinct signatures in degenerative rotator cuff pathologies. *Journal of Orthopaedic Research : Official Publication of the Orthopaedic Research Society*.

[158] Kasper D. M., Moro A., Ristori E. (2017). MicroRNAs establish uniform traits during the architecture of vertebrate embryos. *Developmental Cell*.

[159] Moro A., Driscoll T. P., Boraas L. C. (2019). MicroRNA-dependent regulation of biomechanical genes establishes tissue stiffness homeostasis. *Nature Cell Biology*.

[160] Millar N. L., Gilchrist D. S., Akbar M. (2015). *MicroRNA29a* regulates IL-33-mediated tissue remodelling in tendon disease. *Nature Communications*.

[161] Chen Q., Lu H., Yang H. (2014). Chitosan inhibits fibroblasts growth in Achilles tendon via TGF-*β*1/Smad3 pathway by miR-29b. *International Journal of Clinical and Experimental Pathology*.

[162] Lu Y. F., Liu Y., Fu W. M. (2017). Long noncoding RNA H19 accelerates tenogenic differentiation and promotes tendon healing through targeting miR-29b-3p and activating TGF-*β*1 signaling. *FASEB Journal : Official Publication of the Federation of American Societies for Experimental Biology*.

[163] Chen L., Wang G. D., Liu J. P. (2015). miR-135a modulates tendon stem/progenitor cell senescence via suppressing ROCK1. *Bone*.

[164] Liu Q., Zhu Y., Zhu W., Zhang G., Yang Y. P., Zhao C. (2021). The role of microRNAs in tendon injury, repair, and related tissue engineering. *Biomaterials*.

[165] Rinn J. L., Chang H. Y. (2012). Genome regulation by long noncoding RNAs. *Annual Review of Biochemistry*.

[166] Prensner J. R., Chinnaiyan A. M. (2011). The emergence of lncRNAs in cancer biology. *Cancer Discovery*.

[167] Zuo C., Wang Z., Lu H., Dai Z., Liu X., Cui L. (2013). Expression profiling of lncRNAs in C3H10T1/2 mesenchymal stem cells undergoing early osteoblast differentiation. *Molecular Medicine Reports*.

[168] Zhu L., Xu P. C. (2013). Downregulated lncRNA-ANCR promotes osteoblast differentiation by targeting EZH2 and regulating Runx2 expression. *Biochemical and Biophysical Research Communications*.

[169] Loewer S., Cabili M. N., Guttman M. (2010). Large intergenic non-coding RNA-RoR modulates reprogramming of human induced pluripotent stem cells. *Nature Genetics*.

[170] Pittenger M. F., Mackay A. M., Beck S. C. (1999). Multilineage potential of adult human mesenchymal stem cells. *Science (New York, N.Y.)*.

[171] Prockop D. J. (1997). Marrow stromal cells as stem cells for nonhematopoietic tissues. *Science (New York, N.Y.)*.

[172] Wang Y., Xu Z., Jiang J. (2013). Endogenous miRNA sponge lincRNA-RoR regulates Oct4, Nanog, and Sox2 in human embryonic stem cell self-renewal. *Developmental Cell*.

[173] Keniry A., Oxley D., Monnier P. (2012). The *H19* lincRNA is a developmental reservoir of miR-675 that suppresses growth and *Igf1r*. *Nature Cell Biology*.

[174] Lustig O., Ariel I., Ilan J., Lev-Lehman E., De-Groot N., Hochberg A. (1994). Expression of the imprinted gene H19 in the human fetus. *Molecular Reproduction and Development*.

[175] Goshen R., Rachmilewitz J., Schneider T. (1993). The expression of the H-19 and IGF-2 genes during human embryogenesis and placental development. *Molecular Reproduction and Development*.

[176] Chen Q., Hou D., Suo Y., Zhu Z. (2022). lncRNA XIST prevents tendon adhesion and promotes tendon repair through the miR-26a-5p/COX2 pathway. *Molecular Biotechnology*.

[177] Andersson T., Eliasson P., Hammerman M., Sandberg O., Aspenberg P. (2012). Low-level mechanical stimulation is sufficient to improve tendon healing in rats. *Journal of Applied Physiology (Bethesda, Md. : 1985)*.

[178] Eliasson P., Andersson T., Aspenberg P. (2012). Achilles tendon healing in rats is improved by intermittent mechanical loading during the inflammatory phase. *Journal of Orthopaedic Research : Official Publication of the Orthopaedic Research Society*.

[179] Eliasson P., Andersson T., Aspenberg P. (2009). Rat Achilles tendon healing: mechanical loading and gene expression. *Journal of Applied Physiology (Bethesda, Md. : 1985)*.

[180] Morita Y., Watanabe S., Ju Y., Xu B. (2013). Determination of optimal cyclic uniaxial stretches for stem cell-to-tenocyte differentiation under a wide range of mechanical stretch conditions by evaluating gene expression and protein synthesis levels. *Acta of Bioengineering and Biomechanics*.

[181] Pelled G., Snedeker J. G., Ben-Arav A. (2012). Smad8/BMP2-engineered mesenchymal stem cells induce accelerated recovery of the biomechanical properties of the Achilles tendon. *Journal of Orthopaedic Research : Official Publication of the Orthopaedic Research Society*.

[182] Majewski M., Betz O., Ochsner P. E., Liu F., Porter R. M., Evans C. H. (2008). *Ex vivo* adenoviral transfer of bone morphogenetic protein 12 (BMP-12) cDNA improves Achilles tendon healing in a rat model. *Gene Therapy*.

[183] Lee J. Y., Zhou Z., Taub P. J. (2011). BMP-12 treatment of adult mesenchymal stem cells in vitro augments tendon-like tissue formation and defect repair in vivo. *PLoS One*.

[184] Jelinsky S. A., Li L., Ellis D. (2011). Treatment with rhBMP12 or rhBMP13 increase the rate and the quality of rat Achilles tendon repair. *Journal of Orthopaedic Research : Official Publication of the Orthopaedic Research Society*.

